# The Development and Clinical Applications of Oral Arsenic Trioxide for Acute Promyelocytic Leukaemia and Other Diseases

**DOI:** 10.3390/pharmaceutics14091945

**Published:** 2022-09-14

**Authors:** Lynn Chin, Cyrus R. Kumana, Yok-Lam Kwong, Harinder Gill

**Affiliations:** Department of Medicine, School of Clinical Medicine, University of Hong Kong, Hong Kong 999077, China

**Keywords:** oral arsenic trioxide, acute promyelocytic leukaemia, acute myeloid leukaemia, lymphoma, autoimmune disorders

## Abstract

Appreciation of the properties of arsenic trioxide (ATO) has redefined the treatment landscape for acute promyelocytic leukaemia (APL) and offers promise as a treatment for numerous other diseases. The benefits of ATO in patients with APL is related to its ability to counteract the effects of PML::RARA, an oncoprotein that is invariably detected in the blood or bone marrow of affected individuals. The PML::RARA oncoprotein is degraded specifically by binding to ATO. Thus ATO, in combination with all-trans retinoic acid, has become the curative treatment for ATO. The multiple mechanisms of action of ATO has also paved the way for application in various condition encompassing autoimmune or inflammatory disorders, solid organ tumours, lymphomas and other subtypes of AML. The development of oral formulation of ATO (oral ATO) has reduced costs of treatment and improved treatment convenience allowing widespread applicability. In this review, we discuss the mechanisms of action of ATO, the development of oral ATO, and the applications of oral ATO in APL and other diseases.

## 1. Introduction

Oral arsenic has for centuries been a traditional medicine for diseases ranging from infections to malignancies. Traditional Chinese medicine employed arsenic formulations for over 5000 years as cancer therapeutics. In the eighteenth century, arsenic was used in western medicine for treating malaria, and later further popularized as therapy for a wide array of diseases including leukaemia, parasitosis, chronic skin conditions, syphilis and asthma [1,2,3]. With the development of modern therapeutics, arsenic gradually faded into oblivion.

**Figure 1:**
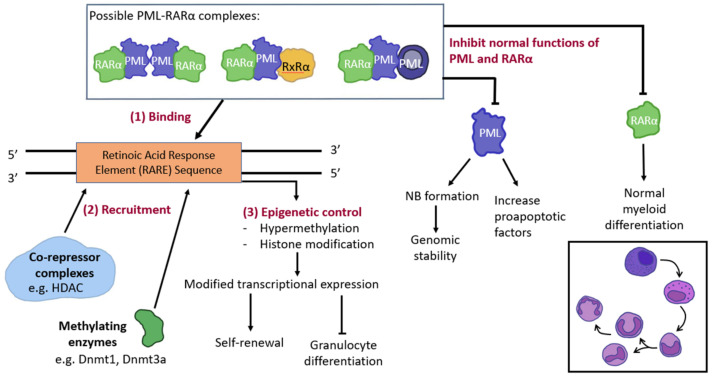
Pathogenesis of acute promyelocytic leukaemia (APL). The formation of various PML::RARA complexes due to the translocation between chromosomes 15 and 17 t(15;17)(q24;q21), which bind to the retinoic acid response element (RARE). The binding of PML::RARA to RARE leads to the recruitment of co-repressors and methylating enzymes (e.g., DNMT1 (DNA (cytosine-5)-methyltransferase 1) and DNMT3A), resulting in epigenetic silencing, inhibition of granulocyte differentiation, accumulation of abnormal promyelocytes and increasing self-renewal. Formation of PML::RARA complexes also inhibits normal functions of PML and RARA such that PML is unable to cause cell apoptosis and tumour suppression via nuclear body (NB) formation. RARα ability to allow localization with RxRα resulting in transcriptional differentiation.

The interest in arsenic has been rekindled because of its therapeutic role in acute promyelocytic leukemia (APL), a subtype of acute myeloid leukaemia (AML) [4]. APL accounts for 10–15% of AML cases and is characterized by t(15;17)(q24;21), which leads to fusion of the promyelocytic leukaemia (*PML*) gene on chromosome 15 with the retinoic acid receptor alpha (*RARA)* gene on chromosome 17 [5]. The resultant PML::RARA fusion protein interferes with promyelocytic differentiation (Figure 1) [4]. Treatment of APL with chemotherapy carries a high mortality rate, because of bleeding from a characteristic coagulopathy. With the realization that APL cells can be induced to differentiate by all-*trans* retinoic acid (ATRA), inclusion of ATRA into frontline treatment has improved the outcome. However, about 35% of newly-diagnosed APL patients might still die during treatment with ATRA-chemotherapy [4]. The outlook is significantly changed with the use of arsenic trioxide (ATO), which is currently incorporated into induction treatment with ATRA with or without [6].

Apart from APL, ATO has also shown promise in other malignant and non-malignant diseases [7]. This review will focus on the clinical development of oral ATO and how this process has increased the current understanding of disease therapy.

## 2. The Discovery and History of Arsenic Trioxide

Arsenic has had a complicated folklore and medicinal history as both a toxin and a remedy. Arsenic was first described as a treatment for skin ulcers in the times of Hippocrates (460–370 BC) and in the ancient Chinese medical text HuangDi NeiJing (263 BC). As alluded various forms of arsenic were used in China for over 5000 years. Arsenic first appeared in Western in the 18th century. It was first patented in 1771 by Thomas Wilson for the treatment malaria and agues. Subsequently, Dr Thomas Fowler from Edinburgh produced a 1% solution of potassium arsenite, known as “Fowler’s solution” for treating severe fevers and leukocythemia [8,9]. From the 1830s to the 1930s, this oral arsenic solution was predominantly used for the management of syphilis, parasitic infestations, chronic skin conditions and asthma [10]. In Haematology, oral arsenic was first reported in the treatment of chronic myeloid leukaemia from the 1860s to 1920s in Germany and Boston City [9,11]. This phased out following World War II with development of alkylating chemotherapy and radiotherapy. With its connection to the West under the British Colonial rule, oral Fowler’s solution, commonly also known as “liquor arsenicals” was produced in Queen Mary Hospital, Hong Kong until the mid-1950s when its use as an anti-leukemic agent was replaced by chemotherapy and radiotherapy [12].

**Figure 2:**
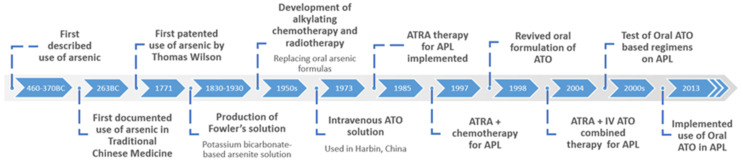
Timeline of advances in APL treatment and the development of oral arsenic trioxide (ATO).

The era of oral ATO was ushered in by the development of a crude intravenous (i.v.) formulated solution of ATO (AiLing number 1), which resulted in a complete remission (CR) rate of 87% [13]. Pure i.v. ATO solution was first used in Harbin, China in 1973. Its mechanism, pharmacokinetics and clinical efficacy was extensively published in 1996 [8]. Subsequently, a pure intravenous solution of ATO was developed in Harbin and used to treat APL patients, which resulted in a CR rate of 73% in newly-diagnosed cases and 52% in relapsed cases [11]. Further studies of i.v. ATO (dosage of 10–15 mg/day) in various centres showed a similarly high CR rates of 72% to 91% in APL patients [12,13,14]. Zhang et al., reported the use of intravenous ATO (10 to 15 mg/day) and showed promising effects in treating APL with further approval by Trisenox (a FDA approved intravenous ATO chemotherapy drug to treat APL) in July 2002 [15,16]. But drug associated adverse events (AE) were noted and include; hyperleukocytosis in 75.6% of patients (183/242 APL patients) leading to APL differentiation syndrome (APLDS). Clinical trials on 224 APL patients using Trisenox displayed water retention and polyneuropathy [16]. It was suggested by Zhang et al., occurrence is related to dosage toxic events and most notably is a leading cause of changes in cardiac activity and GI disturbances due to the effects seen predominately in standard dose than the low dose patient cohort [15]. In Figure 2, the advancements in APL treatment strategies and oral ATO development is summarised.

In the early 2000s, an oral formulation of ATO was developed in Hong Kong for the treatment of APL [10]. Pharmacokinetic studies showed that oral ATO achieved comparable systemic bioavailability as i.v. ATO [10,11]. The use of oral ATO in treating APL in the relapsed/refractory setting was highly effectively, and successfully replaced ATRA -chemotherapy (e.g., ATRA-idarubicin or ATRA-daunorubicin) and hematopoietic stem cell transplantation (HSCT) as the salvage treatment of these patients [5,13]. Due to the success of ATO, nanotechnologies have been developed for the delivery of ATO [17]. In particular nanoparticle formulations such as liposomes mediating endocytosis, polymers (DSPE-mPEG) to prolong treatment and decrease non-specific targeted toxicities and hollow porous silica nanoparticles to enhance permeability and targeted uptake by the mitochondria [17]. But further research still needs to be done on the efficacy of ATO in the treatment of both haematological malignancies and solid tumours with the promising nanotechnologies. The current clinical trials focusing APL management using oral ATO will be discussed in depth later in this review.

## 3. Antileukaemic Mechanisms of Arsenic Trioxide

### 3.1. Caspase Induced Apoptosis

PML is a putative tumour-suppressor protein that plays important roles in mediating apoptosis. Via its closedly spaced cysteine residues, PML binds to nuclear bodies (NB) leading to enhanced interaction with caspases to activate apoptosis, thereby allowing downstream maintenance of DNA-associated pathways [14]. Fusion of PML to RARA disrupts the NB binding sites, so that PML::RARA is incapable of activating apoptosis. ATO binds to the cysteine residues and recruits PML to NB, thereby restoring its apopotic capacity [18]. In myeloma cell lines in vitro, ATO treatment activated caspases 9 and 3, whereas in adult T cell leukaemia cells, ATO upregulated caspases 8 and 3 by buthionine sulfoxide dependent reduction of glutathione thereby activating apoptosis of PML::RARA [19,20].

### 3.2. PML and Gene Modification

ATO exposure leading to proteasomal degradation of PML::RARA via a SUMOylation/ubiquitin-mediated pathway has been proposed [21,22]. SUMO-1, a ubiquitin-like protein, causes SUMOylation of lysine residues of the PML moiety resulting in binding to NB [21]. In other malignant cell lines, such as gastric cancer and B-cell lymphoma lines, ATO treatment directly causes G_1_ phase arrest in a p53 dependent manner [23]. Specifically observed in APL cell lines (NB4) p-53 induction and p27^KIP1^ regulation of tumour development driver genes, *Bcl*-2 and *Bax,* can be seen seen where the Bcl-2/Bax complex is translocated to the mitochondria after ATO treatment, causing cell cycle inhibition [24]. Subsequently, apoptosis of PML::RARA occurs due to an increase in mitochondria dissipation and reactive oxidative species (ROS) penetration [18].

The human homolog of Daxx (hDaxx), a nuclear protein, has been associated with cells containing enhanced sensitivity to *FAS*, a death-related gene [25]. FAS activation results in upregulated caspase activity and JUN N-terminal kinase (JNK) pathway [25]. Interestingly, Daxx colocalises with PML oncogenic domains (PODs), which are associated with PML-NB interactions. ATO promotes the accumulation of hDaxx, thereby increasing the sensitivity of cells to FAS-regulation transcription and the sequestration of PML [25].

Another mechanism of targeted therapy towards PML is via SUMOylation of the PML protein [26]. SUMOylation is an important post-translational regulator of biological activity involved in protein stability and localisation [27]. Protein SUMOylation of PML occurs as a stress induced response after ATO treatment [27]. It is found that degradation of PML requires RNF4 (Ring Finger Protein 4) which is a ubiquitin ligase targetinpoly-SUMO-2/3 (small ubiquitin-like modifier-2/3)-modified proteins and promotes PML ubiquitination [26]. RNF4 specifically binds to the short SUMO interaction motifs (SIMs) and RING (Really Interesting New Gene) finger domain of the SUMO conjugates (SUMO-2/3) and causes co-localisation of PML-NB [26]. Therefore, ATO stimulates PML modification by stress-induced SUMOs leading to ubiquitination by RNF4 and the recruitment of proteasome complexes associated to NBs to initiate proteolytic degradation of PML-NB [27,28].

### 3.3. Oxidative Stress

Oxidative stresses induce the accumulation of reactive oxygen species (ROS), which initiate apoptotic signally pathways via damaging proteins, lipids and membranes of cells. Cysteine residues, rich in thiol groups (R-SH), generate disulphide-binding pockets that are targeted by ROS [29]. ATO induces hydrogen peroxide production and iron-dependent ROS synthesis, thereby impacting ROS-sensitive signalling molecules such as TP53, NFKB, and S-nitrosothiols [18]. Furthermore, this leads to reduction of glutathione peroxidase and catalase, which are crucial for cell protection [30]. Therefore, ATO treatment causes a redox imbalance skewing to excessive ROS. Cysteine-rich hubs located on proteins, such as PML::RARA, are susceptible to ROS and hence redox regulation [30]. In addition, current treatment strategies include ascorbic acid, which inflicts a greater decrease of glutathione levels whilst oxidising intracellular hydrogen peroxidase, thus acting synergistically with ATO to increase apoptosis.

Arsenite also enhances flavoprotein-dependent superoxide-producing enzymes (NADPH oxidase), causing DNA strand degradation, nitric oxide production and micronuclei formation [31].

Studies on NB4 cell line have suggested ATO’s ability to modulate 2-Cys peroxiredoxins (PRXs), a group of enzymes involved with the peroxide reaction of H_2_O_2_ (an important metabolite in the production of ROS) and have been found to be associated with leukemogenesis and tumour cell survival [32]. Upon oxidation of a subtype (PRX3), dimerization occurs and positively correlates with ATO treatment induced ROS production, suggesting a relationship of ATO-dependent oxidation of PRX3 [33]. PRX3 is a specific mitochondrial protein, and when activated interacts with mitochondrial electron supply proteins; thioredoxin-2 (TRX2), thioredoxin reductase-2 (TRX-R2), and nicotinamide adenine dinucleotide phosphate (NADPH) [32]. Notably, both TRX2 and TRX-R2 expression are downregulated in ATO treated NB4 cells, subsequently increasing mitochondrial ROS production and increasing cell death [33]. It is also suggested, ATO decreases sulfiredoxin-1 (an intermediary protein preventing hyper-oxidation and activation of PRXs) translocation to the mitochondria [34]. Sulfiredoxin-1 therefore amplifies apoptosis by preventing PRX activation and indirectly inhibiting ERK/NRF2 signalling cascade hence limiting activation of pro-survival genes and antioxidant genes like PRDX1 (Peroxiredoxin 1) [33,34]. Therefore the relationship between ATO’s upregulation of oxidized PRX3 dimer and downregulation of its corresponding antioxidant proteins (TRX, NADPH) could be another mechanism of ATO in ROS initiated PML::RARA cell death.

Another possible mechanism promoting ROS generation is through ATO’s interaction with arsenite (+3 oxidation state) methyltransferase (AS3MT). After ATO intake, ATO is hydrolysed to inorganic arsenic and transported into the liver [35]. With the help of AS3MT the cytotoxic arsenic metabolites are significantly reduced and distributed from the liver to the blood stream and rest of the body [36]. The mechanism if AS3MT is through promoting the conversion of ATO to monomethylarsonous acid (MMA^III^) and then dimethylarsinous acid (DMA^III^) more reactive methylated metabolites and thus increase ROS concentration [36]. AS3MT is a cysteine rich enzyme and interacts with ATO via the S-adenosylmethionine (SAM) binding domain [37]. Upon binding a conformational change initiates methylation of ATO alongside modulation of antioxidant glutathione (GSH) [38,39]. Subsequently, ROS generation is increased by MMA^III^ and DMA^III^ by targeting the mitochondrial electron chain complexes II and IV [40]. The full role of AS3MT pathway influencing biological activity is still scarce and should be further studied, but the insight of AS3MT activity on ATO chemical modification has shown a different light towards the metabolism of ATO and ROS generation.

Contributing to oxygen transport, CD71 is overexpressed in haematological malignancies and solid tumours. CD71, also known as transferring receptor protein 1 (TfR1) is important in the uptake of transferrin-iron complexes and can be used as a biomarker for actively proliferating cells [41]. Clinical findings of trivalent arsenic strong affinity on leukaemia types and may suggest ATO’s possible mechanism targeting the protein ferritin [42].

ATO further increases lipid peroxidation of the mitochondrial membrane by oxidative injury via decrease in glutathione peroxidase activity through nuclear factor erythroid 2-related factor 2 (Nrf2) signalling pathway [43,44,45]. Nrf2 activity can increase antioxidant enzyme, glutathione-S-transferases (GSTs) and GSTs link to inhibition of mitogen-activated protein (MAP) kinase. Therefore inhibition of Nrf2 indirectly increases protein interactions of c-Jun N-terminal kinase 1 (JNK1) and apoptosis signal-regulating kinase-1 (ASK1), a MAP kinase, to interact in cellular survival and death signals (apoptosis signal-regulating kinase) [46]. Thus, the interference of the mitochondria stability, may be ATO’s main source of ROS production [47].

An anticancer drug (+)α-tocopheryl succinate (α-TOS) has been studied alongside ATO and ATRA and shows potential additive effects after ATO treatment [48,49]. The mechanism of α-TOS occurs by destabilization of the mitochondria and increase in ROS production [48]. Mitochondria destabilization occurs by the modulation of the mitochondrial respiratory chain involving complexes regulating ionic transportation and ionic balance [49]. The dissipation of the mitochondrial membrane potential leads to ROS production and cytochrome release via the use of α-TOS [48]. Leading to PML::RARA partial degradation in NB4 cells. As the reported study focused on the use of α-TOS as an alternative APL treatment and enhanced the effects of ATO treatment after 24 h, more studies regarding the role of mitochondrial respiratory chain complexes could be done with ATO.

### 3.4. Other ATO Targeted Protein Interactions

Heat shock protein (Hsp) modulation has been closely interlinked with ATO oxidative stress, with noticeable upregulation of signal transduction cascades such as MAP kinase, JNK and p38 [50]. Following arsenic exposure, there are elevated levels of the stress protein heme-oxygenase-1 (HO-1) with subsequent increase of heme to biliverdin conversion, and carbon monoxide and ferrous ion generation [51], which constitutes a pro-inflammatory state [18,51].

One study on Hsp60 shows ATO can promote p53 and survivin protein degradation by disrupting Hsp60-p53 and Hsp60-survivin complexes [52]. Degradation of survivin relieves its apoptotic inhibitory role and the release of p53 limits its ability as a tumour suppressor protein when bound to Hsp60 [53,54]. Via the proteasomal pathway, 26 S proteasome degrades p53 and survivin and noticeable with p53 colocalising in one part of the cell [52].

Another possible ATO targeted protein is Pin1, a commonly overexpressed protein in haematological malignancies and mediates nuclear translocation and post translational modification of proteins [55,56]. ATO directly and non-covalently binds to the active site of Pin1 (a peptidyl-prolyl cis/trans isomerase (PPIase)) resulting in inhibition and degradation of APL cells. In leukaemia, ATO uptake decreases Pin1 ability to cause cell proliferation through the activation of NF-κb pathway and therefore inhibits thrombosis. Simultaneously ATRA increases uptake of ATO via aquaglyceroporin 9 (AQP9) upregulation [56].

ATO directly binds to the Gli (glioma-associated oncogene homolog) proteins thus influencing Gli transcriptional effectors [57]. Initially found to be amplified in malignant glioma cells, numerous studies have found haematological malignancies show an increased Hh/Gli pathway activation with aberrant expression of hedgehog (Hh) observed [58]. Hh is important for cell proliferation and differentiation [58,59]. Hh associated ligands (Sonic Hedgehog (SHh), Patched receptor (Ptch1, Ptch2), Smoothened receptor (Smo)) activates Gli transcription factors [58]. Gli-1 and Gli-2 proteins also modulate transcriptional activity downstream by binding to promoter sites of PTCH1, PTCH2 and GLI1 [58,60].

Other proteins have been suggested such as enzymes involved in glycolysis, hexokinase-2 (HK2), in which metabolic instability can result mitochondrial malfunction [61]. ATO directly binds to Cys256 and Cys704 of the HK2 protein where it is localised to the mitochondrial outer membrane [62]. It is often overexpressed in many cancers and is seen to be a repressor of apoptosis [63]. In addition, post transcriptional modifying proteins with rich cysteine zinc finger/ring finger domains are favourable to modulation by arsenic. Examples include CASP14L, MAPK11 and SIRT4 [62].

### 3.5. Myeloid Differentiation

**Figure 3:**
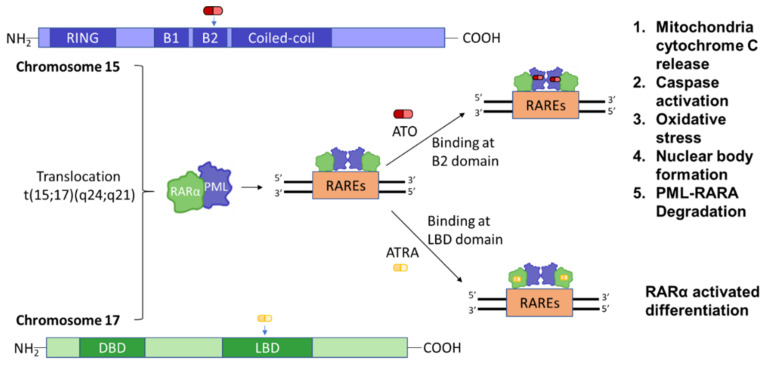
Synergistic effect of all-trans retinoic acid (ATRA) and arsenic trioxide (ATO) on PML::RARA. A translocation event results in the oncoprotein PML::RARA binding to RAREs (Retinoic Acid Response Element (RARE) Sequence). ATO binds to the B2 domain of PML and ATRA binds to the LBD domain of RARA, resulting in ta synergistic effect. DBD: DNA-binding domain; LBD: Ligand-binding domain.

As potent differentiating agents, ATO and ATRA act synergistically in removing the inhibition of myeloid differentiatiation caused by PML::RARA [64]. ATRA and ATO target both PML and RARA for an inter-dependent effect (Figure 3). ATRA induces APL blast differentiation due to characteristic changes in nuclear structures; whereas ATO restores normal PML localization and percievable PML::RARA degradation [18]. A study in vivo in mice suggested ATRA or ATO alone achieved a 35 to 39% survival, whereas ATRA followed by ATO increased the survival to 70–80% [65]. One mechanism of combined ATRA/ATO treatment is a sustained differentiation of cells by demethylation of GC-rich regions located on transglutaminase 2 (TGM2) and retinoic acid receptor beta (RARB) promoter region, thereby preventing histone modification and restoring normal myeloid maturation [65]. ATRA and ATO bind RARA and PML respectively, with the latter interaction leading to PML-NB degradation [66]. Due to ATO-initiated PML::RARA degradation, studies conducted on RA-resistant and RA-sensitive APL patients observed elimination of PML::RARA and decreased abundance of t(15;17) in both mature and immature myeloid cells, suggesting ATO alone could indirectly lead to differentiation [66].

Another potential mechanism regarding activation of mitotic machinery has been linked with ATO induction of abnormal mitotic spindle localisation and formation [67]. Arsenite has previously been studied by binding to cysteine residues of the GTP-binding site of tubulin [68]. Tubulin makes up microtubules important for mitotic spindle formation and chromosome movement [69]. Arsenite indirectly attenuates cell mitosis by modifying centrosome behaviour [67]. It is also an inhibitor of HSP70 and HSP90 which influences tubulin polymerisation and thickens microtubules resulting in aggregation and therefore a prolonged S phase and [70]. In relation with ATO‘s role, time-lapsed observation of ATO-arrested mitotic cells show dissipated mitotic spindle localisation causing random misplacement within the cell [67]. Suggesting similar mitotic ability as arsenite though more correlative studies need to be done. In addition, ATO indirectly modulates Rho and Rho GTPase activity through Rho-associated protein kinase (ROCK) cascade [71]. Through irregular centrosome positioning and production, the activity of microtubules is limited and thus chromosome stability at mitotic level is altered [67]. At prometaphase stage, the effect of microtubular polarity is decreased by altered spindle formation thus limiting cell proliferation [67,69].

PP2 (a pyrazolopyrimidine compound) is a type of Src family protein tyrosine kinase inhibitor and influences APL cell differentiation [72,73]. Inhibitors of the Src family protein alone inhibit cell migration, invasion and apoptosis of non-small lung cancer cells and have shown synergistic effects with ATRA and ATO in NB4 APL cells [72,73]. Though, it has been studied in lung cancer the regulation of PI3K/Akt signalling pathway is demonstrated and can be linked with ATO’s role in apoptosis in APL cases [72,73].

Furthermore, ATO is seen to interact with phosphatidylinositol 4,5-bisphosphate (PIP2). PIP2 influences and indirectly interacts with β-tubulin and mitotic spindle formation [67]. PIP2 is produced by the phosphorylation of phosphatidylinositol-4-phosphate and phosphatidylinositol-5-phosphate with PIP5KI and PIP4KII respectively [74]. Dysregulation of PIP2 directly alters the phospholipid component of cell membranes and can cascade to a wide range of cellular processes [74]. Including the role of PLC (an enzyme hydrolysing PIP2 to form IP3 and DAG) and activation is influence by PIP2 concentration [74]. The subtype PIP4KIIγ has been found to modulate ATO activity which could suggest an indirect role of PIP2 and phospholipase C- γ1 (PLC-γ1) [75]. PLC-γ1 contains the pleckstrin homology (PH) domain and directly interacts with β-tubulin thus centromere formation [74,75]. Therefore, though more studies of ATO’s relationship with PLC-γ1 mediate β-tubulin modification could be done, ATO can mediate spindle activity and limit mitosis of unfavourable cells via PIP2 activation [67].

MicroRNAs (miRNAs) are often involved in post transcriptional regulation of gene expression involved in normal haematopoiesis task such as myeloid differentiation, proliferation and apoptosis [76]. miRNA’s function by binding and inhibiting their target mRNA’s 3′ untranslated regions (UTRs) [77]. In 2013, a group first found ATO has the ability to down regulate two miRNA’s, miR-766 and let-7d, which subsequently increased expression of caspase-3 and Bax, key regulators of ATO-induced degradation [78]. Whereas a more recent study on miR-139-5p shows ATO can increase expression and subsequently modulate early haematopoiesis [79]. A Increased miR-139-5p leads to an increased expression of MNT and its ability to form a heterodimer with MAX [80]. The MNT/MAX complex interacts with the PI3 kinase signalling cascade thereby supressing differentiation of myeloid progenitor cells by inhibiting proapoptotic genes [79]. Thus, dysregulation of the miRNA network is a strongly correlated with ATO-induced apoptosis of APL cells.

## 4. Clinical Development of Oral Arsenic Trioxide in the Management of Acute Promyelocytic Leukaemia

With memories of the Fowler’s solution, we revived an oral ATO formulation or the “modern” liquor arsenicals in 1998. We were the first to produce the 1 mg/mL oral-ATO solution and to show that the mean plasma and blood area-under-the-curve (AUC) were 99% and 87% of that achieved with i.v. ATO [10]. At 48 h following oral-dosing, the intracellular arsenic levels were 270% higher than the corresponding plasma values [10] An important concern of i.v. ATO is corrected QT (QTc) prolongation and ventricular arrhythmias. Due to lower peak plasma arsenic levels following oral-ATO, significant QTc prolongation or ventricular arrythmias are rare except in patients with severe pre-existing cardiomyopathies [81,82,83]. With plasma and intracellular arsenic level monitoring, we were also capable of administering oral-As_2_O_3_ at a lower dose safely in patients with end-stage renal failure on peritoneal or haemodialysis [84,85]. We also showed that after an oral administration, meaningful cerebrospinal fluid (CSF) levels of arsenic were achieved implying its benefit in the prophylaxis or treatment of central nervous system (CNS) disease [86,87]. CSF and plasma arsenic levels were linearly correlated with CSF arsenic levels at 17.7% the plasma levels [86]. We were the first to demonstrate excellent long-term outcome of APL treated with oral-ATO-based regimens [88,89,90]. In a 15-year prospective follow-up study in 73 patients with relapsed APL, idarubicin (6 mg/m^2^/day for 5 days) plus oral-ATO (10 mg/day), all-trans-retinoic acid (45 mg/m^2^/day) and ascorbic acid (1 g/day) (AAA) for 42 days resulted in a 100% molecular remission rate [88]. Following second complete remission (CR2), 2 monthly cycles of idarubicin (6 mg/m^2^/day for 3 days) plus AAA for 7 days followed by 12 cycles of AAA maintenance (given for 2 weeks every 2 months for 2 years) resulted in 5-year and 10-year overall survival (OS) of 79.5% and 67.3% respectively [88]. Importantly this was achieved without hematopoietic stem cell transplantation (HSCT) in CR2. This shows that prolonged AAA maintenance is an effective post-remission strategy following CR2, obviating the need for HSCT, a procedure still considered a standard in many placed around the world in APL following CR2. We then moved AAA forward as post-remission maintenance following CR1 which resulted in a 5-year leukaemia-free survival (LFS) and OS of 90% and 97% respectively [89,90]. More recently, we incorporated AAA (given for 42 days) into frontline induction for newly diagnosed APL with daunorubicin (50 mg/m^2^/day for 3 days) followed by 2 cycles of consolidation with daunorubicin (50 mg/m^2^/day for 2 days) and cytarabine (100 mg/m^2^/day for 5 days) and 2 years of AAA maintenance. Both LFS and OS were 100% at 5 years [89]. In patients aged 70 or above or those with medical co-morbidities, chemotherapy was omitted and patients were treated with an entirely oral regimen comprising 42 days of AAA and no relapses were observed so far [89]. With AAA-based regimens, outcome of both newly diagnosed and relapsed APL were independent of the conventional risk scores. With LFS plateauing 2 years after completion of maintenance both in CR1 or CR2, long-term molecular monitoring is not necessary beyond 2 years after completion of AAA maintenance following CR1 or CR2 [88,89]. In our oral-ATO studies, both short-term and long-term cardiac safety was confirmed. QTc prolongation, ventricular arrhythmias and cardiac failure were not observed Drug-induced transaminitis (Grade 1–2: 30.8%; Grade 3–4: 25.8%) were all reversible with transient dose interruptions [89]. Ascorbic acid was used in the AAA regimen due to its synergism with ATO that has been shown in-vitro and clinically [90,91,92]. Acyclovir prophylaxis is used universally in all patients on oral-As_2_O_3_ due to risk of herpes zoster that was demonstrated in our earlier studies [93]. Differentiation syndrome (DS) occurred in 25.8% and 12% in newly diagnosed APL and APL in first relapse (R1) respectively with no induction deaths observed. With early recognition, cytoreduction and the use of dexamethasone, interruption of oral ATO or ATRA was not necessary in our studies. We are currently testing frontline induction with AAA in APL (ClinicalTrials.gov Identifier: NCT03624270) in a risk-adapted manner incorporating a chemotherapy-free approach.

Within Europe a 5-year randomized study between October 2007 and January 2013 was conducted on APL patients receiving either combined therapy of ATRA and ATO or ATRA and chemotherapy (Italian-German APL 0406 trial) [94,95]. Study cohort includes newly-diagnosed, low- or intermediate-risk patients ranging from 18–71 years old and have been morphologically diagnosed based on the French-American-British criteria for APL diagnosis. In total 276 patients were randomly assigned to either combination therapy. A total of 129 were randomly assigned to ATRA-ATO arm and 137 assigned to ATRA-chemotherapy arm. The purpose of this 5-year study was to observe whether ATRA-ATO is similar or better than ATRA-chemotherapy for patients located within the Italian, German and AMLSG (Acute Myeloid Leukaemia study) group. Using Trisenox (i.v. ATO) as a reference dosage has been standardized to 0.10 or 0.15 mg/kg/day ATO and 45 mg/m^2^ ATRA until CR or up to a maximum of 60 days (approved safety dosage on an adult person). In total after third consolidation, 115 patients went through PCR in the ATRA-ATO arm with 100% being PCR negative for the PML::RARA transcript and out of 119 patients in the ATRA-chemotherapy arm 117 were PCR negative [94,95]. Complete remission rates for the ATRA-ATO arm showed 100% and 95% for ATRA-chemotherapy arm [96]. In the ATRA-chemotherapy arm a significant number of patients observed haematological toxicities of grade 3 or 4 neutropenia (85% compared to 15% in ATRA-ATO arm) and thrombocytopenia (76% compared to 23% in ATRA-ATO arm) of more than 15 days at third consolidation [96]. Other non-haematological toxicities were observed more frequently in the ATRA-chemotherapy arm including GI toxicities and cardiac function abnormalities. However elevated liver function tests were more frequent in ATRA-ATO arm (44%), but could be resolved after temporary discontinuation of ATRA or ATO, in comparison with 3% in ATRA-chemotherapy arm [96]. To conclude, event free survival in the ATRA-ATO group is 98.3% with the ATRA-chemotherapy arm showing 86.6% [96]. Relapses were more predominantly in the ATRA-chemotherapy arm with 15 relapsing at a median of 14 months and 2 relapses in the ATRA-ATO arm at 22–27 months [96]. Overall, both treatment arms showed an improved survival and relapse risk in patients but in the ATRA-ATO arm there is a reduced haematological and non-haematological toxicities associated with treatment observed. Thereby signifying the role of ATO as a nanotechnology in the treatment of APL.

## 5. Applications of Arsenic Trioxide beyond Acute Promyelocytic Leukaemia

### 5.1. NPM1-Mutated AML

Interestingly, AML with mutated *NPM1* gene has some resemblance to APL with the presence of a mutant oncoprotein *NPM1* [97]. Furthermore, several studies have shown that ATRA and ATO treatment results in the selective proteasomal degradation of mutant *NPM1*, nucleolar redistribution of wild-type *NPM1*, reversal of PML nuclear body disorganisation and pronounced apoptosis and/or differentiation [98,99]. These effects may be related to an increase in ROS and TP53 activation [98]. Reduced blasts and/or haematological improvements were observed in clinical trials of ATRA and ATO treatment of elderly individuals with mutant-*NPM1* AML ineligible for chemotherapy [98]. Therefore, the role of ATO in mutant-*NPM1* AML may be analogous to that of APL treatment. These results suggest that a potential oncoprotein targeting strategy for other oncoprotein-linked diseases may be a common mechanism underlying potential efficacy of ATO in these malignancies.

ATO also has the potential to inhibit AKT (protein kinase B) and extracellular signal-regulated kinase 1/2 (ERK1/2) cascade. By inhibition of ERK1/2 there is a reduced phosphorylation at Thr163 site of the myeloid leukaemia cell differentiation protein (Mcl-1) resulting in destabilisation of the protein [100]. In AML, the Mcl-1 protein displays antiapoptotic effects by sustaining development of AML cells [99]. Via the AKT pathway Mcl-1 is also downregulated by the inhibition of GSK-3β phosphorylation of Mcl-1 at Ser159 amino acid site [100]. The combined phosphorylation of Mcl-1 leads to an increase release of caspases and initiates degradation of AML cells similar to the targeted therapy antibody gemtuzumab ozogamicin (GO (CMA-676); Wyeth Laboratories, Mylotarg^®^, Philadelphia, PA, USA) [101].

Additional studies have also been done in regards to ATO resistant AML cells reducing activity of the JAK2/STAT3 pathway [102]. A combination of ATO and ruxolitinib was employed to increase ATO sensitivity. Specifically, G1/S cell cycle arrest by ROS production and downregulation of GSH [102]. The mechanism of ruxolitinib shows synergism in reducing proliferation and metabolic activity of AML cells [102]. Therefore, additional studies in regards to the use of JAK2 inhibitors, ruxolitinib or fedratinib, could be beneficial to prevent ATO resistance and provide a synergistic role in treating diseases.

Due to the availability of oral ATO formulation predominantly in Asian markets a micron-sized oral ATO capsule formulation was developed in the United States called *ORH-2014*. This nanotechnology shows good maximum observed concentration (C_max_) and area under the plasma drug concentration-time curve from 0 to 24 h (AUC0-24) [96]. A total of 12 patients with haematologic malignancies AML (*n* = 4), MDS (*n* = 6) and CMML/MPN (*n* = 2) receiving either 5 mg (*n* = 3). 10 mg (*n* = 6) or 15 mg (*n* = 3) of ATO. Within the trial, ORH-2104 is compared with i.v. ATO (approved dosage of an adult person of 10 or 15 mg) [96]. At 15-days treatment of ORH-2014 at both dosages showed AUC0-24 was better than Trisenox with similar C_max_ indicating rapid dissolution of arsenic [96]. Common drug related AE showed ~25% of patients obtained grade 1–2 nausea, diarrhoea and headache [96]. But low to no noticeable hepatic toxicities were detected, opposing i.v. ATO obtaining in some cases liver toxicities of grade 3 or more [96]. For cardiac toxicities, 1 patient showed grade III QT prolongation but was easily reversed with alternative drugs [96]. ORH-2014 did not record efficacy of ORH-2014 but one patient obtained complete marrow remission at 12 and 27 weeks with a daily dosage of 5 mg [96]. This study shows oral formulations of ATO can become a promising alternative to i.v. ATO due to the absence of common adverse side effects related to high arsenic exposure [96]. But further comparison with other oral ATO formulas need to be done and studying the efficacy on a larger cohort including paediatric patients.

### 5.2. Lymphoma

Mantle cell lymphoma (MCL) is a B cell non-Hodgkin lymphoma with a median survival of ≤3 years. The combinatory regimen of ATO and ascorbic acid was administered for 6 weeks (a time frame common for APL patients) together with chlorambucil to stage III/IV MCL patients, leading to an ORR of 49% (CR: 28%), results that compared very favourably with other drugs typically used in the salvage of such patients [7,10,103,104,105]. The underlying mechanisms appear similar to those previous observed in APL, including up-regulation of pro-apoptotic pathways by activation of caspase activity, TP53 upregulation and suppression of adhesion molecules (NF-kB, IL-6, IL-8 and c-IAP2 mRNA) [104]. Studies had also shown that the combination of ATO with the proteasome inhibitor bortezomib synergistically targeted the 26 S proteasome, increasing ubiquitin-dependent apoptosis and activating NF-kB pathways [104]. Both bortezomib and ATO achieved good rates of cell cytotoxicity at low concentrations, which were further confirmed by in vitro experiments on Mino and Rec-1, bortezomib-resistant MCL cell lines. Hence, ATO in combination with other drugs shows promise as a salvage therapy for late-stage or relapsed/refractory MCL [7].

Another mechanism of ATO is its additive role with the combination of Lenalidomide (a drug for the treatment of multiple myeloma, smouldering myeloma and myelodysplastic syndrome) has the ability to decrease proliferation of the Kaposi sarcoma herpes virus (KSHV) in primary effusion lymphoma (PEL). PEL is a non-Hodgkin B cell lymphoma subset with a rare aggressive onset [106]. At the latent stage of KSHV survival, viral proteins are produced and predominantly resides in B cells with proteins promoting tumour progression [107]. Apoptotic tumour gene, p53, is inhibited by the latent transcript LANA-1 (latency-associated nuclear antigen-1) of the KSHV [106,108]. ATO treatment causes IFNα release of ROS, inhibition of the NF-κB cascade by targeting P-IκBα and nuclear translocation of p65 (an activator of NF-κB pathway) [106]. NF-κB promotes tumour-cell proliferation by preventing cell differentiation and angiogenesis factors (vascular endothelial growth factor (VEGF)) [108,109]. Subsequently, latent viral transcripts of KSHVs were downregulated thus inhibiting further malignancy of PEL [110]. Limiting the release of autocrine growth factors (like NF-κB) and PEL proliferative cytokines IL-10, IL-6 and VEGF are all mechanisms of ATO in treating PEL [106].

### 5.3. Lung Cancer

A common cause of relapse in lung cancer patients is the presence of putative lung cancer stem-like cells (CSC). The malignant phenotype, poor prognosis, and chemotherapeutic resistance have all been attributed to persistence of lung CSCs [111]. A key transcription factor, Glil, helps preserve the lung CSC function by causing the activation of signalling pathways; Hedgehog, Notch, and WNT [112]. Interestingly, ATO might act on lung CSC by suppressing CD133 (found on lung CSC and a marker associated with cancer progression) with resultant downregulation of Glil, N-MYC and GAS1, leading to inhibition of the transcription factors *OCT4* and *SOX2* important for maintenance of lung CSC [113]. ATO also potentially activates tumour necrosis factor (TNF)-families ligands and deplete antioxidants, glutathione and thioredoxin [114,115]. Anti-angiogenesis properties of ATO see decrease in VEGF and cell migration, with a resultant reduction in vascular density [115]. There appears to be a differential effect of ATO on lung cancers, with adenocarcinoma and large cell carcinoma cells less and small cell lung cancer cells more sensitive to ATO [115]. Therefore, additional studies are warranted to support a therapeutic role of ATO in lung cancer [115].

Loss of mitochondrial instability can result in downstream cytochrome c release, caspase 3 activation and finally apoptosis. SH-SY5Y cells are ATO resistant cells and display activity associated with long term use of ATO and inheritably increased levels of Bcl-2 protein [116]. ATO increases in interferon- γ leading to activation of the janus kinase/STAT pathway in the mitochondria and subsequently, STAT3 interacts with Cyclophilin D to regulate the mitochondrial permeability transition pore (MPTP) and increases cytosolic calcium, activation of caspase-2 and release of cytochrome C leading to apoptosis [117,118]. Noted in squamous cell carcinomas (SCLC) a downregulation of X-linked inhibitor of apoptosis (XIAP) and Bcl-2 activity, increases caspase 3, 7 and 9 [111]. Bcl-2 is commonly associate with Bak and the release of Bak activates its proapoptotic properties [119]. In lung adenocarcinoma, E2F1 is downregulated by ATO leading to increased activation of caspase 3 and Bid, and downstream disruption of the cell cycle stages G_1_ to S phases which are modulated by E2F transcription factor 1 (E2F1) [119,120]. Thymidylate synthase, an enzyme involved in DNA synthesis of thymidylate, is downregulated at a protein and mRNA level and ribonucleotide reductase M1 (RRM1), a subunit involved in deoxynucleotide production, is also downregulated in lung adenocarcinoma related ATO treatment [119]. Therefore, ATO mechanism of action targets a wide range of lung CSC transcription, mitochondrial permeability, mRNA transcriptional modification in lung cancer.

### 5.4. Autoimmune Disorders

Multiple sclerosis is an autoimmune disease closely associated with the demyelination of white matter of the central nervous system (CNS) [121]. The resultant loss of the protective myelin sheaths leads to nerve damage and serious neurologic sequelae [121]. Research on experimental autoimmune encephalomyelitis (EAE) mice with CNS injury by simulating myelin-specific T cell activation was used to test the therapeutic role of ATO [122]. EAE mice treated with ATO had attenuated demyelination, reduced inflammation and improved microglial activation [122]. A subsequent decrease in IL2, IL6 and IFN gamma decreased molecule adhesion and leukocyte migration into the CNS [122]. Also, by suppressing damage due to activation of the FAS pathway, ATO may also have potential therapeutic roles in autoimmune lymphoproliferative syndrome and other rheumatic diseases [122,123].

ATO has also been tested on rheumatoid arthritis where there is an autoimmune attack targeting the joints. To further attenuate the diseases, angiogenesis is required for pannus development [124]. ATO has the ability to target the *TSP*-1–*TGF*-β1-*CTGF*-*VEGF* pathway by targeting *TGF*-β1. Firstly, at low doses arsenic upregulates TGF-β1 expression but at high doses ATO decreases TGF-β1 expression levels [125,126]. Clinical studies of previously non-treated rheumatoid arthritis have shown targeted approach of ATO on Treg/Th17/Th1/Th2 cells [127]. Treg cell generation prevents Th17 cell differentiation, decreases STAT3 mRNA transcription factor and subsequently increases the regulatory protein FOXP3 which is key in modulating the activity of genes associated with the immune system [127]. Upregulation of regulatory T cells (Tregs) therefore modulates *TGF*-β1 expression [127]. To show ATO mechanism, experiments in combination with *TGF*-β1 inhibitors show restored differentiation of the bone marrow signifying the importance of eliminating *TGF*-β1 role in stimulating vessel proliferation [127,128]. Via the TGF-β/Smad pathway, miRNA-21 inhibits cell proliferative and proangiogenic genes *Smad7*, *PDCD4*, *PTEN*, and *Spry* [126]. ATO also limits angiogenesis by targeting NADPH oxidases, by modulating AT_1_R protein expression and downstream NO production [126].

### 5.5. Glioblastoma

Glioblastoma is a malignant brain tumour made up of glioma cells, a central nervous system neoplasm, originating from glial cells [129]. Genetic mutations associated with ATO resistance and relapse is seen with *SLIT2* and may be involved with PML-NB formation [130]. In glioblastoma cells, PML promotes cell migration of stem cells and neoplastic cells [130]. Therefore, epigenetic status of *SLIT2* results in modulation of Slit proteins and regulation of cell cycle proteins TP53, TP73 and CDKN1A which subsequently effects cell migration and may have an indirect role of PML-NB binding [131]. In relation with APL, high WBC of 10 × 10^9/L is associated with low expression of *SLIT2* and ATO resistance [131]. Though this is studied predominantly in glioblastoma cells and hormone derived diseases (e.g., kidney, breast and ovarian cancer), the link between SLIT2 and PML-NB formation is well studied. Therefore, as there is a link towards PML-NB related cancers, including haematological cancers, more studies focusing on SLIT2 should be done to fully understand ATO mechanism of action.

Treatment on U87MG glioma cell lines and patient-derived primary S1 glioblastoma cells have seen ATO suppression of miR182-5p subsequently increasing Argonate-2 (AGO2)-gene silencing of Sestrin 2 (*SESN2* protein coding gene) [132]. Sestrin 2 is a stress-inducible metabolic protein which promotes tumour growth through the inhibition of key mitochondrial enzyme AMP-dependent protein kinase (AMPK) [133]. Upon cytotoxic events, the sestrin (SESN) protein group are upregulated to protect normal cells from further damage, but under cancerous circumstances can become proapoptotic by limiting production of ROS through perodoxin and Nrf2 signalling cascade [133]. In addition, ROS generation is correlated with ATO ability to phosphorylate DNA damaging proteins (ATM, ATR, 53BP1, γ-H2AX and Mer11) targeting telomere length can induce apoptosis via p53 and p21 modulation [134,135]. Thus, ATO role in inducing anti-oxidant responses is key in treating glioma tumours.

## 6. Conclusions

The increasing clinical knowledge and understanding of molecular pathways have enabled the redevelopment of ATO, with the most spectacular success observed in the treatment of APL. With the knowledge of the mechanisms of ATO in apoptosis, regulation of key genetic sites and activation of redox-sensitive pathways, other oncoprotein-related diseases may potentially be amenable to ATO treatment. Finally, the availability of an oral formulation of ATO hold much promise in long-term treatment of these diseases.

## Data Availability

Not applicable.
